# Tuberculosis contact-tracing among Syrian refugee populations: lessons from Jordan

**DOI:** 10.1186/s13031-018-0164-y

**Published:** 2018-07-16

**Authors:** Edouard Hosten, Mandana Mehta, Emmanuel Andre, Khaled Abu Rumman, Dimitri Van der Linden

**Affiliations:** 1 Paediatric Infectious Diseases, General Pediatrics, Cliniques Universitaires Saint-Luc, Université catholique de Louvain, Brussels, Belgium; 2 Fill that Gap, Brussels, Belgium; 3 Department of Microbiology and Immunology, Laboratory of Clinical Bacteriology and Mycology, KU Leuven, Leuven, Belgium; 4 National Tuberculosis Program, Chest Diseases Directorate, Amman, Jordan

**Keywords:** Syria, Refugees, Contact-tracing, Tuberculosis

## Abstract

**Background:**

In response to the influx of displaced Syrians since 2011, the Jordanian National Tuberculosis Program (NTP) implemented a specific Tuberculosis (TB) reduction strategy, including contact-tracing (CT). Contacts of all refugees diagnosed with pulmonary TB (PTB) were registered by the International Organization for Migration and screened for active & latent TB infection (LTBI) in 6 NTP centres.

The objectives of this study were to assess prevalence of active TB and LTBI, risk factors for LTBI as well as program performance.

**Methods:**

We performed a retrospective study among contacts (*N* = 481) of all PTB cases diagnosed between March 2011 and May 2014 (*N* = 76). CT was performed using verbal screening of TB-related symptoms, tuberculin skin test (TST) and chest X-ray.

**Results:**

LTBI was diagnosed in 24.1% of contacts tested with TST while active TB was diagnosed in 2.1% of contacts. Main risk factors for positive TST included smear-positive index case (IC) (OR: 6.33) and previous TB infection in the family (OR: 4.94). Among children, the risk of LTBI was higher when their IC was a care-giving female (OR: 2.83). Prevalence of active TB was two times higher in children under five (U5 s) (5.3%) compared to adults (2.5%).

**Conclusion:**

We found a high prevalence of active TB and LTBI among contacts of PTB cases in the Syrian refugee population, emphasizing the urgent need for host countries to implement CT strategies for refugees. Our results underscore the vulnerability of U5s and contacts of smear-positive IC highlighting the need for specific actions focusing on those groups.

## Background

### Tuberculosis in displaced communities

Tuberculosis (TB) continues to be a major infective cause of mortality and morbidity on a global scale. Despite a recent slow decline in TB incidence and a reduced fatality rate (45% reduction between 1990 and 2013), 9.6 million people in the world developed the disease in 2014 [1]. Simultaneously, forced displacement affects a rising number of people worldwide with well documented public health effects such as excess mortality and morbidity due to communicable diseases [2]. Current scientific literature and guidelines consider TB as a potential major infectious threat in displaced communities. While more than 85% of refugees come from high burden countries [3], Kimbrough et al. showed that displaced populations faced an increased burden when compared to reference populations [4]. Others have found that TB could represent an important cause of death in different refugee camps [5, 6]. The harmful consequences of TB on these populations should therefore not be underestimated and international guidelines recommend the implementation of control programs when certain stability criteria are met [3].

**Fig. 1:**
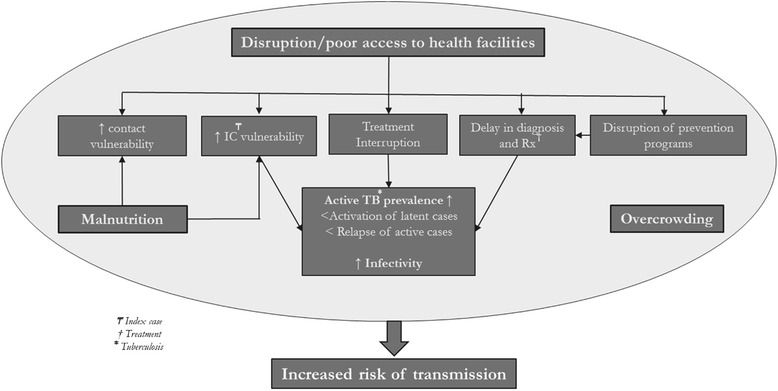
Impact of population displacement on TB transmission

One of the principle impacts expected of forced displacement on TB epidemiology is an increased risk of transmission that can be explained by several factors (Fig. 1). First, population displacement and resettlement in camps or (peri)urban settings result in increased population density which in turn, directly influences the spread of airborne diseases such as TB [7–9]. Second, displacement often induces disruption of and poor access to health facilities, which may impact TB transmission in different ways. There may be delays in diagnosis and treatment of new cases or interruption of on-going chemotherapies, thereby increasing the prevalence of untreated TB and emergence of resistant TB [10]. Disruption of prevention programs reduces public knowledge of TB transmission modes and thereby increases the risk of contagion. Eventually, displacement and poor access to health facilities induce a general increase in comorbidities, including malnutrition, which represent risk factors for development of active TB and increase contact vulnerability [11]. Given this context, the World Health Organization (WHO) and the United Nations High Commissioner for Refugees (UNHCR) invite humanitarian actors to develop contact-tracing (CT) activities when implementing TB public health strategies among displaced populations [3].

### TB & Syrian refugees in Jordan

The violent civil war that is on-going since March 2011 has forced hundreds of thousands of Syrian families to leave their homes. Among the 3 millions Syrian citizens officially registered as refugees worldwide [12] in mid-August 2014, 609,000 had found shelter in Jordan.

This massive influx of refugees most likely increased the TB burden in this country of 6.5 million inhabitants. Indeed, albeit Syria achieved important gains in TB prevention with an incidence divided by three between 1990 and 2012 [13], it still remained three times higher than in Jordan prior to the war (18 new cases per 100,000 inhabitants per year vs 5.8 in 2012). Even assuming an unlikely stable TB incidence in the displaced Syrian population, the arrival of Syrian citizens in Jordan theoretically induced 8 extra new cases of TB in the country in 2012, 85 in 2013 and 105 in 2014 considering the median number of refugees each year [12]. Following the interruption of health services in Syria BCG coverage dropped from 90% in 2005 to 81% in 2014 according to WHO estimates [14].

**Fig. 2:**
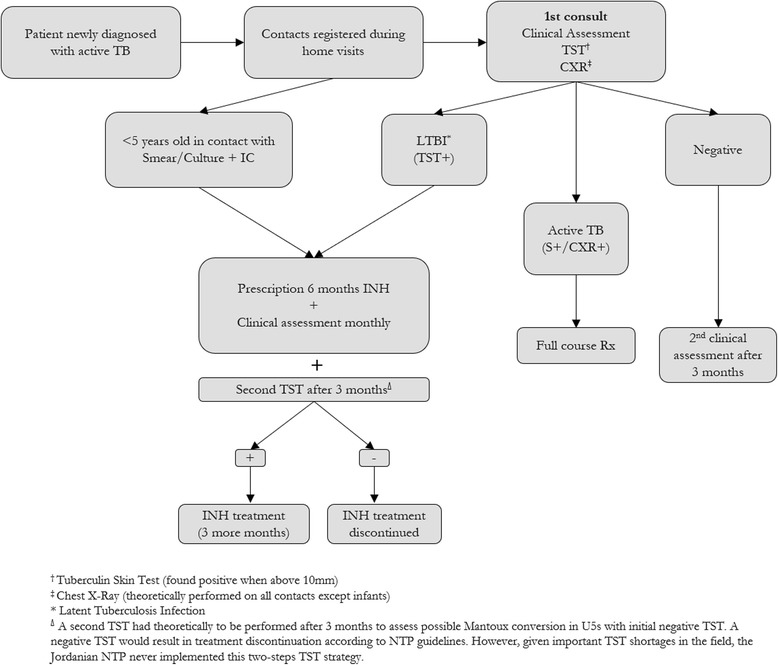
Overview of the implemented CT program

Aware of this challenge, the Jordanian National Tuberculosis Program (NTP) decided to implement a specific TB public health strategy, in coordination with the International Organisation for Migration (IOM), UNHCR and WHO [15]. Alongside screening, diagnosis and treatment, this strategy included the implementation of a CT program, described in Fig. 2.

## Objectives

This paper aims to study the outcomes of the CT program implemented by the Jordanian NTP among Syrian refugees. It will focus on the following three main objectives: i) to assess prevalence of active TB and latent TB infection (LTBI) among different age groups in the contact population; ii) to determine if certain index case (IC) or contact characteristics are risk factors for LTBI and finally iii) to evaluate whether a CT program in refugee populations can be properly implemented and perform to adequate international standards.

## Methods

### Study participants & data collection

This retrospective study includes all Syrian refugees diagnosed with pulmonary TB (PTB) registered by the Jordanian NTP between January 2011 and May 2014 (*N* = 76) as well as their close contacts screened by NTP and IOM (*N* = 481).

Data collection took place between the 1st of August and the 15th of September 2014. Patients’ files, including contact information, were collected in the relevant NTP centres and centralized in Amman for translation. Additional information in English regarding contacts was provided by the IOM in an Excel file. Twenty- four variables were extracted and recorded for statistical analysis. The final sample included only cases with complete records.

Contact age was a continuous variable in the original database, but was recoded into three age groups (< 5 years old, 5-15 years old and older than 15 years old) to facilitate comparison. The composition of age groups among contacts was close to that of the general Syrian refugee population: Children under 5 years old (U5 s) represented 17.0% of our contact population vs 16.9% of all refugees; children between 5 and 15 years old composed 30.0% of contacts vs 34.8% of children between 5 to 17 years in the global refugee population [12]. Age had not been recorded for 34 contacts.

### Definitions

The following definitions were used in this study:

- *Index case* (IC) is defined as any PTB case among the Syrian refugee population treated within a NTP centre in Jordan. Cases of extra-pulmonary TB (EPTB) were excluded.
- *Contact* is defined as “a person who shared the same enclosed living space for one or more nights or for frequent or extended periods during the day with the IC during the 3 months before commencement of the current treatment episode” [16].
- *LTBI* is defined as having evidence of M. tuberculosis infection by immunologic tests (Tuberculin Skin Test (TST) > 10 mm after 48–72 h in both children and adults) in a patient in whom active TB was excluded.
- *Active TB case* is defined either as *bacteriologically confirmed TB* (whereby a biological specimen was positive by smear microscopy (Ziehl-Neelsen stain) or culture) or *clinically diagnosed TB* (whereby the criteria for bacteriological confirmation are not fulfilled but the following three clinical conditions are present: cough for more than three weeks; no response to non-TB antibiotics and a chest radiograph compatible with TB). PTB and EPTB cases in contacts were considered when calculating active TB prevalence in this group.
- The *female caregiver* category includes mothers, grandmothers and aunts, often involved in daily childcare in the Middle East.
- *Previous TB history in the family* is defined as having another member of the family who developed active PTB in the past two years but who is currently not living in the same household as the IC.
- *Median time interval between diagnosis and screening* was defined as the number of days between the diagnosis of PTB in the IC and the contact's first medical consult.
- *Performance indicators* were defined using the objectives for CT programs recommended by the Centre for Disease Control and Prevention (CDC) (Table 5).
- Regarding *treatment outcomes*, we used the following definitions [17]:
  - *Cured*: A pulmonary TB patient with bacteriologically confirmed TB at the beginning of treatment who was smear- or culture-negative in the last month of treatment and on at least one previous occasion.
  - *Treatment completed*: A TB patient who completed treatment without evidence of failure but with no record to show that sputum smear or culture results in the last month of treatment and on at least one previous occasion were negative, either because tests were not done or because results are unavailable.
  - *Not evaluated*: A TB patient for whom no treatment outcome is assigned. This includes cases “transferred out” to another treatment unit as well as cases for whom the treatment outcome is unknown to the reporting unit.

### Statistical methods

Statistical calculations were carried out using SPSS for Windows, version 21.0. A threshold of 95% was used to define statistical significance.

Only contacts who underwent a TST were included in the calculations of risk factors for LTBI, after exclusion of active TB cases. Univariate analysis was performed to assess associations between different possible risk factors and TST positivity. Multivariate analysis was carried out, using binary logistic regression, to explore associations between positive TST and categorical variables for which a significant association was found in univariate analysis. Sex and age were also forced in the model. Results of both uni- and multivariate analysis are expressed as odds ratios with 95% confidence intervals. Odds ratios were considered significant if the confidence interval excluded 1. Exploring associations between the same variables and active TB in contacts could not be performed due to the small number of available observations.

## Results

### Screening tests

Contacts were listed for 71 out of 76 notified TB cases (93.4%), resulting in the evaluation of 481 contacts. Median number of screened contacts per IC was 6 (Table 1). Following a clinical evaluation, 40.3% of patients had a chest X-Ray (CXR) (*N* = 194), 15% a TST (*N* = 72) and 32.4% benefited from both tests (*N* = 156). Fifty-nine of all registered contacts (12.3%) were thus not subject to any test beyond a clinical examination, i.e. 5.2% of contacts aged under 15 years and 17.7% of adults (Table 2). Regarding median time interval between diagnosis and screening, 245 contacts out of 279 for whom screening dates were registered (87.8%) were clinically assessed within 120 days (Table 2). No data regarding contacts HIV, BCG or malnutrition status were recorded.

**Table 1:** Characteristics of Index Cases

IC characteristics	Male	Female	All
Total	52	24	76
Mean age (years)	39.8	34.2	38
	N (% of all IC)	N (% of all IC)	N (% of all IC)
Previous history			
Familial	8 (10.5)	4(5.3)	12 (15.8)
Personal	9(11.8)	2(2.6)	11(14.5)
Familial + Personal	2(2.6)	3 (3.9)	5(6.6)
None	33 (43.4)	15(19.7)	48 (63.2)
PPD			
Positive	10 (13.2)	1 (1.3)	11 (14.5)
Negative	2(2.6)	1 (1.3)	3(3.9)
Unknown	40 (52.6)	22 (28.9)	62 (81.6)
Sputum			
Positive	21 (27.6)	11 (14.5)	32(42.1)
Negative	30 (39.5)	13 (17.1)	43 (56.6)
Unknown	1 (1.3)	0	1 (1.3)
Culture			
Positive	20 (26.3)	10 (13.2)	30 (39.5)
Negative	21(27.6)	8 (10.5)	29 (38.2)
MOTT	0	1 (1.3)	1 (1.3)
Unknown/Not performed	11 (14.5)	5 (6.6)	16 (21.1)
Case finding			
Self-referred	18(23.7)	10 (13.2)	28 (36.8)
Referred from private sector	8 (10.5)	3 (3.9)	11 (14.5)
Referred from public sector	8 (10.5)	3 (3.9)	11 (14.5)
Active case finding (IOM)	18(23.7)	8 (10.5)	26(34.2)
Outcome			
Cured	16 (21.1)	6(7.9)	22 (28.9)
Died	3(3.9)	0	3(3.9)
Lost to follow-up	2(2.6)	0	2(2.6)
Treatment completed	20 (26.3)	13 (17.1)	33 (43.4)
Under treatment	11 (14.5)	5 (6.6)	16 (21.1)
Symptoms at diagnosis			
Cough	44(57.9)	21 (27.6)	65 (85.5)
Sputum	33 (43.4)	14 (18.4)	47(61.8)
Fatigue	33 (43.4)	11 (14.5)	44(57.9)
Fever	31 (40.8)	16 (21.1)	47 (61.8)
Night sweats	34(44.7)	14 (18.4)	48 (63.2)
Loss of weight	36 (47.4)	13 (17.1)	49 (64.5)
Loss of appetite	27 (35.5)	13 (17.1)	40 (52.6)
Chest pain	23 (30.3)	8(10.5)	31(40.8)
Co-morbidities			
HIV	0	0	0
Diabetes	2 (2.6)	4 (5.3)	6 (7.9)
Vaccination (BCG scar)			
Yes	14 (18.4)	7(9.2)	21 (27.6)
No	5 (6.6)	3(3.9)	8 (10.5)
Unknown	33 (43.4)	14 (18.4)	47(61.8)
Median number of contacts screened	5	7	6

**Table 2:** Contacts characteristics and completion of screening tests

	< 5	5 to 15	> 15	Not mentioned	All
Gender	N (% of all contacts)				
Male	40 (8.3)	74 (15.4)	105(21.8)	18(3.7)	237 (49.3)
Female	36(7.5)	60 (12.5)	132(27.4)	16 (3.3)	244 (50.7)
All	76 (15.8)	134 (27.9)	237 (49.3)	34(7.1)	481 (100)
Time from diagnosis to contact screening					
Available data (N)	54	83	126	16	279
Investigations performed before 120 days(%)	43 (79.6)	77 (92.8)	109 (86.5)	16 (100)	245 (87.8)
Median time interval in days	12	7	6	4	7
Complementary exams performed	N (% among age group)				
Nothing	3 (3.9)	8 (6.0)	42 (17.7)	6 (17.6)	59 (12.3)
CXR only	12(15.8)	36 (26.9)	136(57.4)	10 (29.4)	194 (40.3)
PPD only	28 (36.8)	39 (29.1)	3 (1.3)	2 (5.9)	72 (15.0)
CXR + PPD	33 (43.4)	51 (38.1)	56 (23.6)	16(47.1)	156 (32.4)
Total	76 (100)	134 (100)	237(100)	34 (100)	481 (100)
TST results					
Not performed	15	44	178	16	253
≤ 10 mm	45	71	43	14	173
> 10 mm	16	19	16	4	55

### Active TB

Ten cases with active TB disease were diagnosed, resulting in a global TB prevalence of 2.1% among contacts (Table 3). Prevalence was two times higher among U5s (4 cases/76, i.e. 5.3%) compared to adults (6 cases/237, i.e. 2.5%). No active cases were found in children between 5 and 15. 2 extra-pulmonary TB cases were diagnosed, including one in the U5 group (lymph node TB) and one in the adult group (spinal TB). In U5s, mothers were the IC in all cases.

**Table 3:** Prevalence of active TB and LTBI among contacts

	Contacts of all IC				Contacts of Smear + IC only			
	All	< 5	5 to 15	> 15	All	< 5	5 to 15	> 15
All contacts (n)	481	76	134	237	210	33	60	87
Active TB (%) (IC 95)	2.1 (1.1-3.9)	5.3 (1.7-13.7)	0	2.5 (1.0-5.7)	3.8 (1.8-7.6)	9.1 (2.4-25.5)	0	5.7 (2.1-13.4)
PTB prevalence (%) (IC 95)	1.6 (0.7-3.3)	3.9 (1.0-11.8)	0	2.1 (0.8-5.1)	2.9 (1.2-6.5)	6.1 (1.1-21.7)	0	4.6 (1.5-12.0)
EPTB prevalence (%) (IC 95)	0.4 (0.1-1.6)	1.3 (0.1-81)	0	0.4 (0.0-2.7)	1.0 (0.2-3.8)	3.0 (0.2-17.5)	0	1.1 (0.1-7.1)
Contacts with TST result available (n)	228	61	90	59	120	28	44	34
LTBI prevalence (%) (IC 95)	24.1 (18.8-30.3)	26.2 (16.1-39.3)	21.1 (13.5-31.2)	27.1 (16.7-40.5)	37.5 (29.0-46.8)	39.3 (22.1-59.3)	40.9 (26.7-56.7)	35.3 (20.3-53.5)

Eight patients presented a pulmonary form of the disease, all except 1 presenting at least one typical TB symptom (cough, loss of weight, night sweats or prolonged fever). The proportion *of clinically diagnosed PTB* among U5s was 33% (1 case/3) and 80% in adults (4 cases/5). All cases of EPTB were clinically diagnosed. All U5s with active PTB had a positive TST. All adults with active TB tested with TST had a positive result (3 cases). Among adults with PTB, 2 cases (40%) had no TST performed and were not bacteriologically confirmed.

Among U5s, 3 patients had completed their treatment while 1 was under treatment at the time of the study. Among adults, 3 patients had completed their treatment and 1 was cured while treatment outcomes were not evaluated for 2 at the time of the study.

### LTBI

Fifty-five cases of LTBI (24.1%) were diagnosed among contacts tested with TST (Table 3). Prevalence was found significantly higher (*p*-value < 0.001) in contacts of smear-positive ICs (37.5 (29.0-46.8) % - 45 cases on 120 contacts tested) than in contacts of smear negative IC (9.3 (4.8-16.8) % - 10 cases on 108 contacts tested).

The difference in mean number of screened contacts was not found to be significant between households in which LTBI was diagnosed (8.63) and those in which it was not (5.46).

### INH preventive therapy

52 Fifty-two out of the 55 contacts diagnosed with LTBI (94.5%) received isoniazid (INH) therapy, i.e. 93.8% of U5s, 100% of 5 to 15 year old and 87.5% of adults. This coverage dropped to 28.6% in U5s with a negative TST but in contact with a bacteriologically confirmed IC (6 contacts out of 21). Altogether, INH was provided to 58 contacts out of the 76 patients (76.3%) who should have benefited from INH prophylaxis according to national guidelines (Table 4).

**Table 4:** Proportion of contacts who were prescribed INH when required

Contacts diagnosed with LTBI								U5 s in contact with bacteriologically confirmed PTB				All contacts requiring prophylaxis			
< 5		5-15		> 15		All		Any TST		TST -		< 5		All	
N	Under INH (%)	N	Under INH (%)	N	Under INH (%)	N	Under INH (%)	N	Under INH (%)	N	Under INH (%)	N	Under INH (%)	N	Under INH (%)
16	15 (93,8)	19	19 (100)	16	14 (87,7)	55	52 (94,5)	31	16 (51,6)	21	6 (28,6)	36	21 (58,3)	76	58 (76,6)

### Risk factors for LTBI

Following univariate analysis, risk factors for positive TST in all contacts and all age categories except adults, included smear- and culture-positive IC as well as previous TB history in the family (Table 5). Furthermore, having a female caregiver as IC also represented a significant risk factor in all children under age fifteen.

**Table 5:** Risk factors of LTBI among contacts in univariate analysis

	All *N* = 221 OR (CI95)	< 5y *N* = 57 OR (CI95)	5-15y *N* = 90 OR (CI95)	0-15y *N* = 147 OR (CI95)	> 15y *N* = 56 OR (CI95)
IC characteristics					
Gender (female)	1.61(0.87-2.97)	2.48(0.76-8.07)	1.68(0.60-4.66)	1.91(0.89-4.12)	0.37(0.11-1.27)
Personal History	0.69(0.30-1.59)	0.81(0.22-3.00)	0.21(0.03-1.68)	0.52(0.25-1.42)	1.31(0.28-6.02)
Family History	4.94(2.58-9.48)	3.81(1.11-13.09)	21.71(5.55-85.00)	9.66(3.95-23.64)	0.61(0.18-2.10)
Smear +	6.33(298-13.41)	4.24(1.23-14.64)	31.15(3.93-247.08)	8.7(3.33-22.73)	3.32(0.91-12.05)
Culture +	6.99(3.29-14.84)	3.81(1.11-13.09)	26.07(3.29-206.30)	7.47(2.87-19.46)	3.32(0.91-12.05)
Contact characteristics					
Gender(Female)	0.92(0.50-1.69)	1.05(0.33-3.33)	1.16(0.42-3.21)	1.10(0.62-1.96)	0.47(0.14-1.51)
Age					
< 5y	1.25(0.63-2.47)	–	–	–	–
5-15y	0.71(0.37-1.33)	–	–	–	–
0-15y	0.84(0.45-1060)	–	–	–	–
> 15	1.29(0.65-2.56)	–	–	–	–
Relationship (IC is ...)					
Father	–	1.29(0.39-4.32)	2.96(1.00-8.74)	2.1(0.94-4.68)	–
Mother	–	1.88(0.51-6.94)	1.05(0.33-3.33)	1.32(0.56-3.11)	–
Brother/sister	–	0	0	0	–
Female caregiver	–	3.11(0.94-10.27)	2.83(1.00-8.00)	2.97(1.36-6.48)	–
Husband/Wife	–	–	–	–	0.48(0.14-1.70)
Son/Daughter	–	–	–	–	0.57(0.09-3.76)
Father/Mother	–	–	–	–	1.06(0.30-3.69)

Following multivariate analysis, smear-positive IC and previous history of TB in the family were found as independent risk factors for having a positive TST in all age groups above the age of 5 (Table 6).

**Table 6:** Risk Factors of LTBI among contacts in multivariate analysis

	All *N* = 221 OR (CI95)	<5y *N* = 57 OR (CI95)	5-15y *N* = 90 OR (CI95)	0-15y *N* = 147 OR (CI95)
IC characteristics				
Family History	4.38(1.96-9.82)	3.24(0.76-13.82)	9.49(2.14-42.17)	6.35(2.39-16.90)
Smear +	6.01(2.44-14.79)	9.69(0.87-107.7)	16.89(1.37-208.1)	7.1(1.94-25.96)
Relationship				
Female caregiver	–	1.65(0.42-6.44)	2.84(0.67-12.12)	2.00(0.78-5.16)

## Discussion

### Prevalence of active TB

The prevalence of active TB among all contacts included in our study was 2.1% and 3.8% when only smear-positive IC were considered. Two systematic reviews on contact tracing in low- and middle-income countries [18, 19] found comparable results despite substantial statistical heterogeneity. We found that U5s presented with the highest active TB prevalence, regardless of the smear status of the IC. The difficulties of diagnosing TB in children may distort the calculation of prevalence, however this higher yield in young children, especially infants and children under 2 years of age, is usually explained by a much higher risk of progression to active disease compared to adults [20]. Although the number of contacts U5 found with active TB included in our study was not sufficient to perform a statistical analysis of risk factors for active disease, it is important to note that all presented with their mother as IC. This corresponds to the positive correlation in U5s between having a mother suffering from PTB and developing active disease elaborated in previous studies [21]. This has been explained by mother-child proximity as well as by an increased infectivity in women due to delays in diagnosis and treatment [22]. Interestingly, no active cases were detected in children between 5 and 15 years of age. This could be due to the relatively small number of active TB cases.

### Risk factors for LTBI

Our results showed a positive association between LTBI and being a contact of a bacteriologically confirmed TB case. This is consistent with previous studies in which a correlation between the level of infectivity of the IC and infection among contacts was found [23–25]. Nevertheless, it is important to underline that 10 out of 55 contacts diagnosed with LTBI (18.2%) were living with smear-negative IC.

LTBI in children was also positively associated with a previous history of TB in the family. This could be attributed to increased exposure although no equivalent results were found in the literature. Further studies should address this correlation in order to evaluate the need for systematic screening of previous family history in CT programs.

Univariate analysis showed a positive correlation between LTBI in children and contact with a female caregiver presenting with active TB. Although this association was not found to be significant in the multivariate analysis, the link between latent infection in children and a specific caregiving relationship with IC, particularly mother or aunt, has been reported in other studies and was usually explained by increased intimacy between child and parent [21, 26].

Our results did not show a significant association between average household size and LTBI prevalence. Nevertheless overcrowding has been described elsewhere as a potential risk factor for TB transmission [7–9] while lower socioeconomic status and poor housing conditions have been linked with a higher risk of progression to active disease along other socio-medical factors such as malnutrition [27].

The effects of HIV and other comorbidities such as malnutrition could not be examined in the study population due to lack of data in patient files. The authors highlight the very low prevalence of HIV in pre-war Syria [28] as well as the low levels of global acute malnutrition among Syrian U5 refugees in neighbouring countries including Jordan [29].

### Performance indicators

With 10 cases of active TB and 55 cases of LTBI detected, the CT program implemented by the Jordanian NTP has proven useful. Regarding performance indicators, our results showed that this CT program reached all targets defined by the CDC ([30]; Table 7), with the exception of a slightly inadequate proportion of contacts evaluated for active TB and LTBI (87.7% instead of 90%). The low proportion of contacts evaluated for LTBI may partially be explained by a significant TST shortage between December 2013 and March 2014. This shortage, faced by other health agencies worldwide in 2013-4 [31], compelled the NTP to strictly restrict the use of remaining tests among young children.

**Table 7:** Performance indicators of CT programs

Key Indicator	Objective	Jordan
Proportion of infectious patients with at least one contact listed	90%	93.4%
Proportion of contacts who are evaluated for TB and LTBI	90%	87.7%
Proportion of contacts of contact investigations concluded within 3-4 months after the diagnosis of the index patient	80%	87.8%
Proportion of infected contacts who begin preventive therapy for LTBI	85%	94.5%

Given the high mobility of refugee populations, the fact that contacts were registered for 93.4% of ICs represents a remarkable achievement. Despite the specificities of the studied population these results show that such a program can be successfully implemented among refugees and perform to high standards when integrated into a well structured NTP.

These findings should be linked with a previous study evaluating the public health strategy implemented in Jordan which found that its implementation led to a 40% increase of TB case detection among Syrians in the country [32]. Nevertheless, this positive picture is somewhat darkened by the low proportion of U5s presenting with a negative TST but in contact with a smear-positive case who were prescribed INH preventive therapy. This low coverage seemed to be mainly due to a poor implementation of guidelines especially in one particular NTP centre where only U5s with positive TST received INH.

### Limitations

The first limitation that we faced in this study lies in the suboptimal performance of diagnostic tests used for active TB and LTBI. Poor specificity of TST due to BCG immunization is, for example, often mentioned as a potential source of misdiagnosis of LTBI. A recent study, however, considers that the effect of BCG if received in infancy is very low. A TST > 10 mm can thus be considered as indicative of TB infection in countries where BCG is administered in the neonatal period as in Syria [33]. On the other hand, other factors that lower TB immunity, may cause false-negative TST results. The sensitivity of TST is, for example, lower in children with protein-energy malnutrition [34]. This might have induced an underestimation of LTBI prevalence in our study as nutritional assessments conducted in the Syrian refugee population in Jordan showed significant levels of chronic malnutrition among U5s years in both Zaatari camp (17%) and outside (9%) [35]. As previously mentioned important mitigating data such as HIV status, nutrition status, BCG and TST size were omitted from the records examined for this study. For practical and financial reasons our study was limited to the data collected by NTP and IOM files. This limitation influences the interpretation that can be made on the data collected. Another limitation due to the retrospective design of our study includes incomplete data collection in some patient’s files (eg: age, date of diagnosis…). The at least 4-month shortage of TST that the NTP faced in early 2014 also represents a potential bias that might have led to an underestimation of LTBI prevalence in contacts. Another bias lies in the low bacteriologically-confirmed TB prevalence. As a consequence of limited access to highly sensitive culture test and sometimes imperfect data recording, 5 out of 8 patients notified with pulmonary TB were indeed diagnosed based on clinical symptoms and a recent history of contact with TB. This result should be read in the light of WHO guidelines considering that symptom-based screening strategy may represent a safe and more feasible contact management strategy in resource-constrained settings [36].

Finally the post hoc nature of this analysis prevented us from controlling the quality of the collected information, as the authors noted that the comprehensiveness of medical files greatly varied from one NTP centre to another.

## Conclusions and recommendations

Our results show that a contact-tracing program can be properly implemented in refugee populations and perform to international standards. The estimated prevalence of LTBI and active TB among contacts were significant and similar to previous studies. Based on this study, the following recommendations should be considered:

Given the resource constraints in displacement situations, our findings support the WHO priority for screening U5s and contacts of bacteriologically confirmed IC. Specific attention should moreover be given to children of TB affected mothers as well as to contacts presenting with prior history of TB in the family. However, given the significant TB prevalence among adults found in our study, a contact-screening program should be extended to older children and adults as soon as resources allow.

Further prospective studies should be conducted regarding:

- Assessment of risk factors (including malnutrition, HIV infection and BCG vaccination) for active TB and LTBI in refugee populations with higher TB incidence.
- Cost effectiveness of contact screening programs in refugee populations.
- Symptom-based CT programs assessment in displacement settings, including evaluation of physicians’ clinical evaluation accuracy.
- INH preventive therapy indications and efficiency in displacement situations.

## Abbreviations

BCG: Bacille Calmette Guérin
CDC: Centre for Disease Control and prevention
CT: Contact-Tracing
EPTB: Extrapulmonary Tuberculosis
IC: Index Case
INH: Isoniazid
IOM: International Organization for Migrations
LTBI: Latent Tuberculosis Infection
MOTT: Mycobacteria other than tuberculosis
NGO: Non-Governmental Organisation
NTP: National Tuberculosis Program
PTB: Pulmonary Tuberculosis
TB: Tuberculosis
TST: Tuberculin Skin Test
U5s: Children under 5 years old
UNHCR: United Nations High Commissioner for Refugees
WHO: World Health Organization
